# Implication of Complement System and its Regulators in Alzheimer’s Disease

**DOI:** 10.2174/157015909787602805

**Published:** 2009-03

**Authors:** Martin V Kolev, Marieta M Ruseva, Claire L Harris, B. Paul Morgan, Rossen M Donev

**Affiliations:** Department of Medical Biochemistry and Immunology, School of Medicine, Heath Park, Cardiff University, Cardiff CF14 4XN, UK

**Keywords:** Alzheimer’s disease, neurodegeneration, inflammation, β-amyloid peptide, complement, complement regulators, CD59, complement therapeutics.

## Abstract

Alzheimer’s disease (AD) is an age-related neurodegenerative disease that affects approximately 24 million people worldwide. A number of different risk factors have been implicated in AD, however, neuritic (amyloid) plaques are considered as one of the defining risk factors and pathological hallmarks of the disease. Complement proteins are integral components of amyloid plaques and cerebral vascular amyloid in Alzheimer brains. They can be found at the earliest stages of amyloid deposition and their activation coincides with the clinical expression of Alzheimer's dementia. This review emphasizes on the dual key roles of complement system and complement regulators (CRegs) in disease pathology and progression. The particular focus of this review is on currently evolving strategies for design of complement inhibitors that might aid therapy by restoring the fine balance between activated components of complement system, thus improving the cognitive performance of patients. This review discusses these issues with a view to inspiring the development of new agents that could be useful for the treatment of AD.

## INTRODUCTION

Alzheimer disease (AD) is a neurodegenerative disease that causes changes in brain function. AD usually affects people over the age of 65 years, with a progressive decline in memory, thinking, language and learning capacity. Age is the strongest predictor for the development and progression of AD and with the rapidly aging population, AD clearly poses a major health problem. An estimated 5-10% of the population aged 65 years and over, and 40% of the population older than 85 years of age are likely to be affected by AD [48]. Genetic predisposition accounts for only 5-10% of the risk of AD and is most pronounced in those developing disease at an early age [5, 11].

With the aging population in Western countries and the annual economic cost of AD in terms of both healthcare expenses and lost wages, it is important to understand the disease mechanism and propose effective treatment. AD is diagnosed post-mortem by analysis of brains of patients with dementia. Senile (also called amyloid) plaques and intracellular neurofibrillary tangles (NFT) are considered to be hallmarks of the disease [50]. Senile plaques contain extracellular deposits of amyloid-β protein (Aβ), α-synuclein, ubiquitin, and apolipoprotein E. NFT are intracellular and extracellular aggregates of hyperphosphorylated tau protein and apolipoprotein E [40]. Aβ is derived from the β-amyloid precursor protein (APP) by the proteolytic action of β- and γ-secretases [59]. These enzymes cleave at the N-terminus of Aβ region of APP followed by cleavage at the C terminus producing peptides that are 39 to 42 amino acids in length. The predominant two forms found in amyloid plaques and believed to be associated with disease are 40 and 42 amino acids long. Alternatively the α-secretase cleaves APP within Aβ domain yielding a non-toxic C terminal fragment, followed by its γ-secretase cleavage to a C terminal fragment-γ and a small peptide called P3 that is not considered amyloidogenic. Evidence that Aβ alone is not sufficient for development of AD came from experiments with transgenic mice, which suggested that sustained brain inflammation might be an essential factor in AD pathogenesis. Different inflammatory markers such as activated microglia and astrocytes, elevated levels of various cytokines and complement activation products are found in the AD brain [41]. mRNA for all complement components are detected in brain cells, suggesting local biosynthesis, and levels of C1q and C9 mRNA are markedly upregulated in AD brain [66]. Furthermore, complement proteins are reported to be integral components of amyloid plaques and cerebral vascular amyloid in AD brains. They are found at the earliest stages of amyloid deposition and their activation coincides with the clinical expression of Alzheimer's dementia [1, 8].

Here we review the key role of the complement system in neurodegeneration and prospective strategies for protection of neurons from complement-mediated death.

## THE COMPLEMENT SYSTEM

The pathogenesis of AD is not yet fully clear. Various hypotheses with respect to disease etiology have been proposed (comprehensively reviewed in [40]). It is likely that different primary risk factors synergize to initiate pathology. Due to the exceptionally complex events involved in neurodegeneration in AD and their mutual relationship and control, it is difficult to identify the primary cause of the disease. However, it is obvious that the complement system plays a central role in the amplification of AD risk factors and neuronal death [8]. The complement system is comprised of more than 30 plasma and membrane-bound proteins (Fig. (**1**)) and is activated by three pathways: the classical, alternative and lectin [60]. The classical pathway (CP) is initiated when C1q binds to immune complexes containing IgG or IgM resulting in generation of the C3 convertase. The alternative pathway (AP) does not require antibodies to initiate the cascade, but is triggered by foreign surfaces. This pathway is continuously ticking-over in plasma, due to spontaneous low-level hydrolysis of an internal thioester bond in plasma C3. This ensures an immediate and rapid response in the face of a foreign surface, such as a bacterium. The lectin pathway is activated following recognition and binding of MBL to repetitive carbohydrate patterns on the surface of pathogenic bacteria and viruses. Binding of MBL to pathogens activates the MBL-associated serine protease 2 (MASP-2) that leads to activation of C4 and C2, the pathway proceeds in exactly the same way as the CP [54].

All three pathways result in formation of a C3 convertase. Further C3 is cleaved to C3b which clusters around the initiating convertase and eventually forms an enzyme, the C5 convertase, capable of binding and cleaving the first component of the lytic pathway, C5. C5 is cleaved to C5b, exposing a binding site for the next component C6. By sequential association of the terminal components (C6, C7, C8 and C9) the membrane attack complex (MAC) is formed, this complex is a ‘pore’ which crosses target membranes resulting in osmotic lysis and cell death [14].

Due to continuous low level turnover of complement in plasma, host cells are constantly at threat from ‘accidental’ complement deposition. To survive this threat, cells express complement regulatory proteins which have evolved to protect self cells from damage by homologous complement. Some of these complement regulators (CRegs) are soluble, plasma proteins (C1-inhibitor (C1INH), C4b-binding protein (C4bp), factor H (fH), factor I (fI)), whereas others are expressed on cell membranes (CD46 (also called membrane cofactor protein, MCP), CD55 (also called decay accelerating factor, DAF) and CD59) [29]. CD59 is the only membrane molecule that regulates the terminal complement pathway by binding to C8 and thus preventing formation of lytic MAC [13].

## COMPLEMENT ACTIVATION IN AD

Although viewed for years as an immuno-privileged organ, the central nervous system (CNS) contains and synthesizes many components of the immune system. Both glia and nerve cells in the brain can synthesize complement components [3, 51]. Primary astrocytes and astroglia were shown to produce all of the components of both the AP and CP, and also those proteins involved in the terminal pathway (Fig. (**1**)) [18, 19, 33, 45]. Although CSF levels of MBL have been found to be reduced by 44% in AD patients compared to control subjects [25], there is no evidence for direct involvement of the lectin pathway in activation of complement in AD. Nerve cells can express all of the complement components and should also be considered as an important source of complement [17]. Thus, the local production of complement components in the brain ensures the complete functionality of the complement system without the need of infusion of plasma-derived complement proteins.

Neuritic plaques and NFT comprise the major neuropathological lesions [49]. Neuritic plaques contain degenerating axons and dendrites and extracellular deposits of Aβ that include abundant amyloid intermixed with non-fibrilar forms of this peptide. Aβ has several forms with different peptide lengths. As discussed above, the two major forms Aβ40 and Aβ42 and the short form Aβ28 are predominant in the disease progression. Fibrillar Aβ40 and Aβ42 directly activate the AP and CP *in vitro* by binding to C3 and the globular heads of C1q [24]. It should also be noted that Aβ interactions with C1q lead to increased amyloid aggregation [61], which additionally stimulates activation of the complement system in AD brains. These interactions trigger the covalent binding of C3b to Aβ and subsequent cleavage of C5 into two fragments - C5a which promotes inflammation and C5b which mediates formation of the lytic membrane attack complex (MAC). The MAC then permeabilizes the membrane of the neurons [8], resulting in cellular lysis. Thus, activation of the complement system not only leads to cell death, but also results in formation of proinflammatory molecules C3a, C5a and MAC. The role of these proinflammatory molecules in the progression of AD will be discussed below. In addition to the above, non-fibrillar Aβ42 and the short Aβ28 forms of the amyloid peptide can induce dose-dependent activation of C4. The mechanism of C4 activation is not dependent on C1q, because non-fibrillar Aβ can still activate C4 in plasma genetically deficient in C1q. This may occurs *via* activation of contact/kinin system which has been shown to be markedly activated in CSF of AD patients [4]. Other complement activators, such as amyloid P, C-Reactive Protein (CRP) and Hageman factor, were found in AD lesions and their potential role for the progression of the disease has been reviewed elsewhere [31]. All these data demonstrate the existence of multiple ways for activation of complement cascade in AD, all of which however are triggered either by the amyloid plaques or by non-fibrillar Aβ. Thus, generation of amyloid peptides, both soluble and insoluble, is a major mechanism for activation of complement system in AD brains.

Interestingly, a post-mortem study of AD brains showed markedly upregulated levels of mRNA for complement components in the affected areas of AD brains [66]. In the entorhinal cortex, hippocampus and midtemporal gyrus, with high accumulation of plaques and tangles, up to 80-fold increase in expression of C1q mRNA and also higher C3, C4 and C9 mRNA levels were detected. The levels of mRNA produced from these complement component genes in compartments of the brain affected by disease were even higher than in the livers from the same patients, suggesting local overproduction of the complement components in AD. The detailed molecular mechanisms leading to this local overexpression of complement components in AD brains are yet to be elucidated, however at present it is widely accepted that cytokine expression in the brain stimulates secretion of complement components locally. On the other hand, other studies using RT-PCR, RNA *in situ* hybridizations and immunohistochemical methods show that levels of complement inhibitors in AD are barely increased [2]. Taken together, these facts indicate a major role of complement system activation in the pathology and progression of AD and the C1q molecule takes a central place in activation of complement cascade.

### Can Complement Activation be also Good for Neurons?

There is recent evidence that complement may also play a protective role in AD brain. The most convincing data for a protective role of complement shows that production of C5a results in activation of the neuroprotective mitogen activated protein kinases (MAPK) [35]. In support of this, animals genetically deficient in the complement component C5 were found to be more susceptible to hippocampal excitotoxic lesions [36]. These findings suggest a novel non-inflammatory role for C5a in modulating neuronal responses to excitotoxins. More recently, a protective role of complement in AD was demonstrated in human APP transgenic mice; when complement was blocked by expression of soluble Crry (a rodent CReg with inhibitory activities similar to human CD46 and CD55) [63], mice expressing soluble Crry had a 2-3-fold increase in Aβ accumulation and neuronal degeneration compared to animals that did not express the inhibitor. Crry inhibits C3 activation, and thus prevents generation of C5a. Therefore, the observed effect might also be due to decreased C5a formation. In another recent study, C1q protected cultured primary neurons against Aβ and SAP (serum amyloid P) induced neurotoxicity [39]. The exact mechanism of this protection has not been elucidated but the data suggest that the effect of C1q was not through inhibition of apoptosis in the neurons. However, considering the protective role of C5a, the C1q-mediated defense against senile plaques might be at least in part due to the generation of the anaphylatoxin. This speculation is supported by a recent *in vivo* study in C3 deficient APP transgenic mice that demonstrated accelerated plaque deposition and neurodegeneration compared to C3 sufficient animals [30].

MAC was also demonstrated to have a dual role in neuronal death. Besides the osmotic lysis it could cause, an interesting hypothesis suggests that at sublytic concentrations, MAC can alter the function of neurons by triggering various cellular signaling pathways [8]. This process has not been investigated in neurons; however, a study using Schwann cells as a model system [22] indicated that sublytic MAC rescued cell from apoptosis *via* activation of PI-3 kinase-Akt, followed by MAPK/ERK1 signaling cascade and increased expression of the anti-apoptotic regulator Bcl-xL. Therefore, sublytic MAC detected on Schwann cell *in vivo* during inflammatory neuropathy may facilitate survival of Schwann cell.

In summary, complement may have a dual role in pathogenesis and progression of AD and this should be carefully considered when choosing targets for development of therapeutics.

## ALTERATIONS IN EXPRESSION OF COMPLEMENT REGULATORS IN AD

As reviewed above, activation of the complement system may be both beneficial and detrimental in AD. Therefore, it is of a great importance to understand whether expression of the complement regulators (CRegs) alters as a result of the disease. Astrocytes and microglia in the CNS produce both soluble CReg (clusterin – controls the lytic activity of the MAC, C1INH, fH, fI, C4bp, properdin – a major regulator of the AP which stabilizes C3bBb convertase) and membrane-bound CReg (CD59, CD55, CD46) [18, 58], which ensures a good level of protection against complement activation. However, the same cannot be said for neurons. There is very little information regarding expression of the CRegs in neuronal cells. Studies to date suggest that cultured primary neurons only express low levels of the membrane CReg, CD59 (lower than the astrocytes), and CD55, and secrete only C1INH and clusterin [37, 57]. In NT2-N neuronal cell lines, relatively high expression levels of CD46, CD59 and CD55 are found [38], however, NT2-N are differentiated from NTERA-2 tumour cells (malignant pluripotent embryonal carcinoma) and tumours are known to overexpress these CReg proteins [6, 46]. Our unpublished data demonstrate that neurons differentiated from human neural progenitor cells express very low levels of the three membrane-bound CReg. Hence, neurons are particularly susceptible to complement-mediated death.

Studies on the effect of AD on expression of CRegs in neurons are few in number. Although the importance of this issue is clear, it has been overlooked for years. In a comparative study carried out on RNA samples from AD patients and matched controls, it was reported that C1INH and CD59 expression was slightly upregulated in AD brains [65]. However, this analysis was performed by a semi-quantitative PCR and the reported upregulation of 17% and 12%, respectively, are unlikely to be significant. Moreover, limited overexpression of CRegs in the face of markedly increased expression of complement components C1r (206%), C1s (167%), C5 (135%), C6 (156%), C7 (142%), C8 (408%) and C9 (1530%) implies that neurons in AD brains may be very susceptible to complement attack [32]. In another study it was demonstrated at both protein and mRNA levels that CD59 expression in frontal cortex and hippocampus in AD brains was significantly decreased when compared with normal age matched non-demented individuals [64], supporting the hypothesis that AD brains are particularly vulnerable to complement-mediated neuronal death. Although the data on expression of CRegs in AD are very limited, all studies demonstrated that expression levels of inflammatory mediators are markedly upregulated in AD tissue while those of the complement inhibitors are altered only slightly. This lends further support to the hypothesis that chronic inflammation may be causing neuronal death in AD.

Another mechanism that is likely to contribute to the susceptibility of neurons to complement lysis, which could be spontaneously activated [47, 68], was demonstrated using IMR32 neuroblastoma cells as a model. Loss of all CRegs expressed on the surface of these cells (CD46 and CD59) by shedding into the culture medium was detected in neurons during apoptosis, which for example, could be initiated by the amyloid plaques. Activation of caspases in human neurons does not lead to an immediate and rapid process of cell death but provokes a protracted form of apoptosis [69]. Thus caspase activation in neurons may participate in the long-term overproduction of Aβ, which is consistent with the protracted and age-dependent nature of AD Therefore, the shedding of membrane-bound CReg results in an increased susceptibility to complement-mediated lysis [12]. This might be one of the mechanisms resulting in decreased expression of the membrane-bound CReg proteins in AD brains. Furthermore, as a result of the lost CRegs, these apoptotic cells are likely to activate the complement system, yielding increased local inflammation and contributing to AD related neuronal damage.

## TREATMENT TARGETING THE COMPLEMENT SYSTEM

AD is a complex disease and its management is often challenging. Personality and behavioural changes, and the eventual inability to perform activities of daily living lead to dependence. A cure for AD is still not available and clinicians and caregivers are challenged with caring for an increasingly aging population affected by dementia. The primary goals of treatment are to maximize the patient’s ability to function in daily life, maintain quality of life, slow the progression of symptoms, and treat depression or disruptive behaviors. However, at present, all treatments for AD offer only modest symptomatic relief for periods of between six to eighteen months. Some of the currently used/researched agents for treatment of the disease are considered to prevent/delay AD symptoms, which may be of a great benefit for significantly reducing the number of patient with dementia. There are a number of different categories of drugs used or under research in AD treatment (reviewed in [28]). Here we emphasize therapeutic agents that target the complement system. Until now complement proteins and CRegs have been overlooked as targets for development of drugs for AD treatment, but accumulated data suggest that appropriate modulation of expression and/or activity of proteins controlling the complement cascade would be beneficial for AD patients. Here we review the current, rather unsatisfactory state of this field and, based on the ongoing research, we also speculate on the future developments of complement inhibitor therapeutics.

### Cerebrolysin

Cerebrolysin (CBL) is an anti-inflammatory mixture of neuropeptides (smaller than 10kDa) obtained from porcine brain tissue [44]. It exhibits unique neurotrophic and neuroprotective activity and reduces amyloid burden in animal models. It also improved neurodegenerative alterations in an APP model of AD [43]. CBL regulates the activity of cyclin-dependant kinase-5 and glycogen synthases kinase 3β, which phosphorylate APP, thus reducing levels of phosphorylated APP and accumulation of APP in neuritic processes [44]. This reduces the level of available APP for cleavage by the secretases. Studies in patients with mild to moderate AD have shown that CBL improves cognitive performance [47]. However, considering the complex nature of CBL (produced by standardised enzymatic breakdown of purified porcine brain proteins and containing approximately 25% of low molecular weight peptides (<10 k DA) and a mixture of approximately 75% free amino acids), this therapeutic may well have other features that benefit AD patients. For example, we recently found that CBL protects cultured human neurons differentiated from neural stem cells from complement-mediated lysis by upregulation of expression of CD59 and CD55 (Kolev et al., submitted). This multilevel effect of CBL on neurons, anti-inflammatory, anti-apoptotic and local inhibition of complement on neuronal surface, justify the need of additional well-designed studies, adequately powered, to assess the beneficial properties of CBL.

### Antibody Inhibitors of Complement

Perhaps the most successful soluble therapeutic agents have been antibody-based agents, either full-length immunoglobulin or single-chain Fv (scFv) fragments. The most extensively studied therapeutic antibodies are against C5. Antibodies that bind C5, thereby preventing triggering of the terminal pathway and MAC formation, have been successful in treatment of various pathologies exacerbated by complement activation. Anti-C5 in particular is used for treatment of paroxysmal nocturnal hemoglobinuria (PNH), and is being developed further for treating myocardial infarct (MI) and arthritis [9, 10, 23]. Such antibodies, however, would prevent generation of C5a, which was demonstrated to be of a benefit for clearance of amyloid plaques in animal models of AD. Antibodies have also been generated which target and inhibit other components of the terminal pathway, including C6 and C8. Anti-C8 was shown effectively to inhibit platelet activation in a bypass model, indicating that specific blockade of MAC function might be of therapeutic benefit [42]. Considering the data about the role of complement system in AD (reviewed above), these agents are very promising for management of AD, however, the consequence of such treatment has yet to be tested *in vivo* in AD animal models.

### Inhibitors of Amyloid-Mediated Complement Activation

Amyloid plaques can activate complement *via* binding C1q, resulting in bystander killing of neurons and driving neurodegeneration. Therefore, molecules that could inhibit this interaction might have therapeutic potential on the neurodegeneration reducing its progress. Glycosaminoglycans were demonstrated to have an inhibitory effect on the interaction between C1q and Aβ [56]. A similar structure – activity relationship as on the SAP component – Aβ interaction was shown. This suggests that glycosaminoglycans interfere with the binding site on Aβ for SAP component and C1q.

Another inhibitor of the complement system, C4bp, also was detected in amyloid plaques and on apoptotic cells in AD brain [67]. A very recent study [55] showed that under *in vitro* conditions, C4bp binds apoptotic and necrotic but not viable brain cells (astrocytes, neurons and oligodendrocytes) and limited complement activation on dead brain cells. C4bp also binds Aβ42 peptide directly *via* the C4bp α-chain limiting the extent of complement activation by Aβ. These recent findings suggest that C4bp, respectively C4bp-derived peptides and glycosaminoglycans might be used for therapeutic modulation of complement activation against excessive complement activation in AD brains.

### Soluble Forms of Membrane CReg

In addition to the molecular approach described above, a plethora of soluble therapeutics has been developed which specifically inhibit the complement cascade [34]. Early agents were based on the naturally-occurring membrane-associated CRegs, such as CD46, CD55, CD59 and Complement Receptor 1 (CR1; CD35). Removal of membrane-anchoring domains using recombinant technology generated soluble molecules with the same regulatory functions as the ‘parent’ inhibitor, which could be administered locally or systemically to inhibit complement. The best known of these is a soluble form of CR1 (sCR1), an agent developed nearly 30 years ago and used in the clinic, with limited success, for complement inhibition in MI and cardiopulmonary bypass (CPB) [26, 27, 62]. These ‘first-generation’ agents have been engineered and modified over the years to improve characteristics such as half-life (by introducing antibody Fc domains; [21]), membrane localisation (by using lipid ‘tails’; [52]) and by targeting specifically to sites of complement activation (by using fusion proteins comprising a domain that binds C3 activation fragments, thus localising to the site of complement attack [53]). In the case of AD, it may be beneficial to selectively inhibit the complement cascade. For example, inhibition of the terminal pathway using antibodies that allow C5a formation but prevent MAC formation may prevent neuronal damage but permit the observed protective effects of C5a. Another agent, developed to selectively inhibit MAC formation, but which allow C5a generation, is a CD59-Ig fusion protein [7]. This has been tested in a murine model of laser-induced choroidal neovascularization and shown to have therapeutic effect. This long-lived agent halts the complement cascade at the level of C8. The problem with many of these agents is that their action is systemic, totally blocking the complement cascade. In addition, if they are targeted to the alternative, classical or MBL activation pathways, their long-term use can be detrimental, triggering immune complex disease and permitting bacterial infections as the key role of complement in innate immunity is prevented. Recently we have designed a strategy that renders the active component inactive as a prodrug, which activates at sites of inflammation [20]. Similar approach could be used for activation of prodrugs at sites of chronic inflammation including the CNS in AD.

### Antibody-CReg Hybrid Molecules as Potential Complement Therapeutics in AD

Agents that specifically target complement inhibition to the vicinity of the amyloid plaques would avoid systemic inhibition of complement, and provide enhanced protection to neurons. Previous *in vitro *studies have demonstrated that recombinant techniques can be used to fuse CReg to the carboxy-termini of antibodies or antibody Fab fragments, thus locating the therapeutic action of the inhibitor to the site pre-determined by antibody specificity [68]. If recombinant antibodies to Aβ, or other antigens unique to the plaque, are developed, then specific targeting of CD59 or other agents to sites of disease may be possible. This approach has not been yet studied in animal models of AD, however, we believe it has a great potential in preventing complement-mediated neuronal degeneration and needs further attention.

### Targeting CReg Genes in AD

Recently, we have proposed a new strategy for modulation of expression of membrane-bound CRegs by targeting transcriptional regulators of their genes [15, 16]. We showed that p53 and neuronal-restrictive silencer factor (REST) are potential targets for modulation of expression of CD59 in neurons. We further elucidated the REST-dependent mechanism for suppression of CD59 expression and designed several peptides that could modulate expression of this CReg. One of these peptides up-regulated CD59 in IMR32 neuroblastoma cells and human neurons differentiated from neural progenitor cells by 5-fold at both mRNA and protein levels (Kolev *et al*., submitted). Further analysis of the potential benefits from this overexpression is undergoing, however, it is clear that the CD59 up-regulation results in local suppression of the MAC on neuronal surface, whilst allowing formation of beneficial complement activation products, such as C5a [36]. This approach seems very promising for diseases like AD, although further investigation is needed to clarify potent activators of CReg expression and optimize methods for delivery. DNA-binding sites of transcription factors are highly conserved between species, therefore this approach is translatable to the murine system where adequately powered *in vivo* studies should be carried out.

## CONCLUSIONS

AD is the most common cause of dementia in people over age of 65 and affects more than 24 million people worldwide. This number will increase considerably in the future and will present enormous financial burdens to health care systems. The need for effective treatment is urgent. Current drugs for treating the cognitive impairments in AD are based on neurotransmitter replacement or modulation, which produce mild symptomatic benefit with minimal impact on the disease process. The role of inflammation in AD pathology is increasingly recognized and anti-inflammatory agents suggested as therapy. The complement system and its regulators have been shown to play a key role in neuronal death and represent an attractive target in AD. Preventing complement activation triggered by amyloid plaques by blocking the interaction between complement proteins and Aβ, or by inhibiting the proteolytic cascade of complement activation may reduce neuronal loss. The apparent neuroprotective effect of some complement activation products emphasises the “double-edged sword” nature of complement in this disease. As a consequence, the choice of agent and stage of activation targeted will be crucial for success of anti-complement therapies in AD. Further experiments are needed *in vitro* and in animal models in order to choose drugs which inhibit generation of ‘disease-associated’ complement activities while retaining the ‘beneficial’ ones, thus providing a maximal benefit to AD patients.

## Figures and Tables

**Fig. (1):**
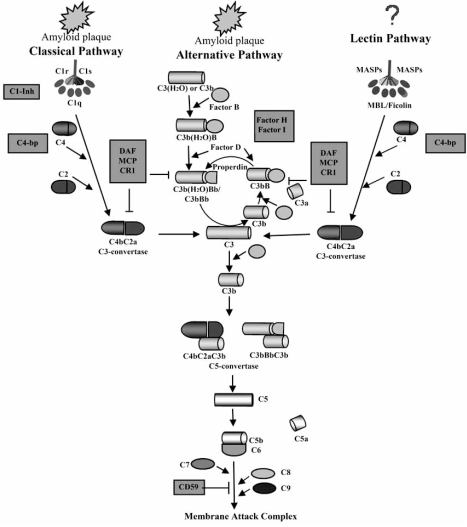
**The complement system in AD.** Binding of C1q and C3 to amyloid plaques can activate the classical and alternative pathways of complement system. No binding of MBL to amyloid plaques and activation of the lectin pathway has been reported. Activation of complement results in cleavage of C3 and C5 by specific enzymes (C3- and C5-convertases) followed by formation of membrane attack complex and cell lysis. Complement is regulated by soluble and membrane bound complement regulators shown in gray boxes.
